# Psychological Violence Against Arab Women in the Context of Social Media: Web-Based Questionnaire Study

**DOI:** 10.2196/27944

**Published:** 2021-08-19

**Authors:** Ahmed Omar Bali, Emad Omer, Kawa Abdulridha, Araz Ramazan Ahmad

**Affiliations:** 1 Diplomacy and Public Relations Department University of Human Development Sulaimani Iraq; 2 Mass Communication Department Ajman University Ajman United Arab Emirates; 3 Sulaimani Polytechnic University Sulaymaniyah Iraq; 4 Department of Administration, College of Humanities University of Raparin Ranya Iraq; 5 Department of International Relations & Diplomacy, Faculty of Administrative Sciences and Economics Tishk International University Erbil Iraq

**Keywords:** psychological, violence, Arab women, social media, feminism, sociology, abuse, oppression, self-esteem

## Abstract

**Background:**

Social media provides women with varying platforms to express themselves, show their talents, communicate and expand their social relationships, and break the shackles imposed by their societies. Theoretically, social media can play a significant role in developing women’s freedom and decreasing social pressures; nonetheless, women continue to face violence during the social media era mainly in the form of psychological violence.

**Objective:**

This study aims to conduct an empirical in-depth analysis of how the digital space, particularly social media, provides men with new opportunities to surveil, restrict, harass, and intimidate feminists in Arab countries.

**Methods:**

This study includes an empirical survey to investigate what Arab women think are the causes and types of violence wielded against them and their perspectives on the impact of that violence. This study used a web-based questionnaire administered through Google Forms (n=1312) with responses from Arab women aged 15 years and above from all Arab countries.

**Results:**

We found that most Arab women feared posting an actual photograph of themselves on their social media accounts and only approximately one-third (490/1312, 37.3%) did so. Most women indicated that they encountered sexual harassment regardless of their age. Furthermore, most women were not aware of the legal aspects of this crime and even those who were aware indicated that they would not press charges for several reasons, including bringing dishonor upon their families, the time-consuming nature of litigation, and fear of revenge.

**Conclusions:**

This study shows that young and less educated women are more vulnerable to abuse from either social media users or being condemned by their families. This has several effects, including lower self-esteem and hesitancy in seeking a job, feelings of mistrust and fear, cynicism, anxiety, depression, and sleep disorders. These issues hold women back from using social media in positive ways and some consider leaving social media.

## Introduction

### Background

Social media provides women with a variety of platforms to express themselves [1-3] and show their talents, communicate, expand their social relationships, and break the shackles imposed by their societies. Theoretically, social media can play a significant role in developing women’s freedom and decreasing social pressures on them [4]; nonetheless, women face high levels of violence on social media, particularly sexual harassment [5-9]. According to an Amnesty International report in 2018 [10], women are subjected to scorn, disrespect, and abuse on the internet, and they encounter a staggering amount of violent or aggressive behavior from their relatives and other individuals. Women in Arab countries use social media with fear and extreme caution. The behavior on the part of others that elicits this fear can be considered violence against women. Moreover, it can be seen as restricting freedom of speech and freedom of thought because of social media’s encompassing role that extends beyond entertainment and leisure and into work and political life as well. Therefore, any kind of violence on social media directed against women negatively affects the physical, mental, financial, and social aspects of their lives.

As a phenomenon, violence against women on social media has not been studied sufficiently to understand this kind of violence better, the reasons for it, and the outcomes of that violence across the Arab region, with the majority of studies focusing on individual countries. This study aims to conduct an empirical in-depth analysis to investigate how the digital space negatively impacts women in Arab countries, using questionnaire surveys. We speculate that social media provides men with new opportunities to surveil, restrict, harass, and intimidate women in Arab countries.

### Conceptual Framework

The intersection of women, feminism, and technology is addressed in many social science disciplines, particularly media and communications political science, sociology, and philosophy [9,11,12]. In order to examine Arab women’s relationship with technology, particularly social media, which theoretically deliberates and empowers women, the dominant feminist approaches must be outlined. However, in this study, the liberal feminist approach will be highlighted to focus on the aspect of women’s self-expression. The conceptual framework considers violence against women across social media, a phenomenon that is more emphasized by radical feminists. Besides the two approaches, *liberal feminist* and *radical feminist*, related studies in Arab countries will be considered.

#### Liberal Feminism and Social Media

The internet, particularly social media, is highly connected to the liberal feminist perspective and ambition. In this context, Megarry argues that “liberal feminism attempts to harness the liberal values of justice, equality, and fairness to fight for women’s rights within the patriarchal state system” [9]. The liberal approach looks positively on the role played by technology, including social media, as a way to enhance women’s lives. Although women face violence across digital technologies, they argue that social media can still bolster women’s interests and rights [13]. The connection between social media and feminism has been examined in several interdisciplinary studies [14-19], in which researchers highlighted the positive aspects of social media that allow women to engage globally [16], contribute to social movements that reflect feminist goals [20], and expand and raise awareness about issues related to sexism, misogyny [21], inequality, and gendered violence [17]. In a similar context, Crossley argues that social media facilitates understanding of women’s issues and enhances feminist ideas across societies [18]. Facebook, Twitter, Snapchat, and Instagram are described as interactive and participatory platforms, which allow for the dissemination of information through the creation, sharing, and consumption of text, audio, and video content [22-24]. From this standpoint, social media enables feminist activists and groups to build and create interactive networks to achieve their goals and shape positive ideas about women and their interests in the face of beliefs and ideas that oppose equality for women.

#### Radical Feminism and Social Media

The radical feminist perspective tends to view the digital space more negatively, seeing women as exposed to abuse and domination of the male perspective [25-27]. Social media enhances radical feminism, which ideologically becomes fragmented and less geographically bound [28]. In other words, radical feminism as a universal approach has been negatively affected because of social media. Even before the emergence of social media, the radical feminist approach rejected the argument that the developments in technology resulted in positive outcomes for women [29-32] because they believe that social institutions are dominated by men [33] and new technology, including social media, is developed in the interest of men [9]. Many studies emphasize this dark side of social media with relation to women. In this regard, Stubbs-Richardson et al [14] show that women are often pathologized or trolled either on the internet or offline and cyberspace is a sexist and gendered space [14] controlled by the logic of power, ideology, and a market-oriented approach. Other studies argue that social media as a commercial platform aims to generate revenue [34]. Other studies, focused on Tinder and dating apps, show that gendered discourses have implications on hidden harassment and abuse of women [35]. From the radical feminist perspective, women face violence and are pathologized or trolled and exploited by men.

#### Status of Arab Women

Like all women, Arab women are affected by cultural factors such as religious beliefs, social systems, education, and the media [36]. Religious beliefs play a remarkable role in social and cultural socialization, working to determine women’s relationship with others, even within the realm of social media [37]. The education system has also portrayed Arab women negatively, and they are directed to deal with men sensitively and with fear [38]. In turn, this results in a tendency for them to adopt conservative values because they still follow the traditional Arab culture where their relationships are limited. While liberal feminists might argue that, in the age of satellite television channels there is opportunity for openness, Arab media tends to focus on women’s bodies as a commodity and promotes women as key to the labor market and in constructing the family and society. Other studies have found that social media has worked to expand the social circles of Arab women [39]. However, they tend to face high levels of violence, with the Arab League releasing a report that Arab women tend to be faced with a lack of real political participation, education, job opportunities, and heath care and are vulnerable to violence as a consequence of regional war and conflict [40]. Similarly, the Economic and Social Commission for Western Asia has released reports that corroborate the Arab League’s findings [40]. Overall, there is a lack of studies on violence on social media against Arab women, with these 2 reports failing to address the issue. Hence, this study seeks to examine this issue to understand the present dynamics and identify possible solutions in a better manner.

### Research Questions and Hypotheses

On the basis of this literature review and our study objectives, 4 questions and 8 hypotheses were developed.

#### Research Questions

1. How do Arab women use social media?
2. Do Arab women use social media freely?
3. How do Arab women face sexual harassment via social media?
4. Do Arab women have legal awareness about sexual harassment on social media?

#### Hypotheses

Along with the research questions shown above, this study tests 8 hypotheses to shed light on the study’s underlying arguments.

1. Level of education is positively associated with posting personal photographs on social media accounts.
2. Age is negatively associated with posting personal photographs on social media accounts.
3. Level of education is negatively associated with being condemned for posting a photograph or about a topic on social media.
4. Age is negatively associated with being condemned for posting a photograph or about a topic on social media.
5. Level of education is associated with being sexually harassed via social media.
6. Age is not associated with being sexually harassed via social media.
7. Level of education is associated with legal awareness about sexual harassment on social media.
8. Level of education is associated with willingness to press charges against sexual harassers.

## Methods

### Methods Overview

This study includes an empirical survey to investigate what Arab women think of the types and causes of violence against them and their perspectives about the impact of that violence. This study used a web-based questionnaire administered through Google Forms (n=1312) with responses from Arab women aged 15 years and older from all Arab countries. The random sampling method is considered one of the best methods since everyone in the research context had an opportunity to participate [41,42]. Consequently, the results represented the population as a whole [43]. Data collection was carried out starting during the last week of October through mid-December 2020. The participants were categorized into five age groups: 15-18 years (134/1312, 10.2%); 19-25 years (608/1312, 46.3%), which was the largest group; 26-35 years (250/1312, 19.1%); 36-50 years (264/1312, 20.1%); and ≥51 years (56/1312, 4.3%). The 19-25–year-old participants comprised the largest group because they are mainly college students, a cohort that actively uses social media. The survey also considered the marital status of the participants. Single women comprised the largest number with 858 (of 1312, 65.4%) participants, married women comprised the second-largest group with 414 (of 1312, 31.6%) participants, and widowed/divorced women comprised the smallest group with 40 (of 1312, 3.0%) participants. The participants were from different educational levels: those who only have reading and writing skills (8/1312, 0.6%), high school students (138/1312, 10.5%), university students (454/1312, 34.6%), those with a bachelor’s degree (446/1312, 34.0%), those with a master’s or PhD degree (244/1312, 18.6%), and activists (22/1312, 1.7%)—referring to those working in political parties and organizations. The demographic characteristics of the study participants are summarized in Table 1. A Spearman correlation model was used to test the aforementioned 8 hypotheses.

**Table 1:** Demographic backgrounds of women (N=1312).

Characteristics			Women, n (%)
**Age (years)**			
	15-18	134 (10.2)	
	19-25	608 (46.3)	
	26-35	250 (19.1)	
	36-50	264 (20.1)	
	≥51	56 (4.3)	
**Education levels**			
	Reading and writing skills	8 (0.6)	
	High school	138 (10.5)	
	University students	454 (34.6)	
	Bachelor’s degree	446 (34.0)	
	Master’s or PhD degree	244 (18.6)	
	Activists	22 (1.7)	
**Marital status**			
	Single	858 (65.4)	
	Married	414 (31.6)	
	Widowed/divorced	40 (3.0)	

### Procedure for Measuring Hypotheses

The data were analyzed by using SPSS (version 22, IBM Corp). Considering the nature and aim of this study, several questions were presented in the questionnaire to measure psychological violence against Arab women through binary scale (“yes/no”) and triple scale (“yes/no/do not know”) modes. “Do not know” was used for some questions, which we predicted that some of the participants would answer with “do not know” to express their views. Furthermore, a triple scale mode “no/somewhat/yes” was used. The “somewhat” response was included to know how many women face sexual harassment or whether they face it rarely. The second model was a Spearman correlation model with Sig. (2-tailed) *P* values, which was processed in SPSS to test the 8 hypotheses through 2 levels of public engagement.

## Results

### Social Media Use by Arab Women

This section examines the first research question, which asked the women who participated to identify their preferred social media platform. Table 2 shows that the majority (770/1312, 58.7%) prefer Facebook and one-fifth (272/1312, 20.7%) prefer Instagram. The third-most popular platform among Arab women is Snapchat, which was used by 128 (9.8%) participants. As shown in Table 3, the user-friendliness of a social media platform was most important to them, which 808 (61.6%) participants selected as the most important reason they chose their preferred platform. The second-most cited reason was cybersecurity, chosen by 138 (10.5%) participants. This reason is important for Arab women, but the majority of them did not highlight this issue because hacking a social media account is not easy and women tend to use social media cautiously (Table 3). In total, 122 (9.3%) Arab women use social media for promotions and personal branding.

**Table 2:** Popular social media platform among Arab women (N=1312).

Social media platforms	Women, n (%)
Facebook	770 (58.7)
Instagram	272 (20.7)
Snapchat	128 (9.8)
TikTok	94 (7.2)
WhatsApp	48 (3.7)

**Table 3:** Reasons for using such social media platforms among Arab women (N=1312).

Reasons	Women, n (%)
Cybersecurity	138 (10.5)
User-friendly	808 (61.6)
Most popular	124 (9.5)
Entertainment	120 (9.1)
Useful for promotion and personal branding	122 (9.3)

### Freedom of Using Social Media by Arab Women

This section examines the second research question, which looks at the freedom with which Arab women use social media. Table 4 shows that most women are cautious about posting photographs of themselves on their social media accounts and only approximately one-third (490/1312, 37.3%) do so. Approximately half (628/1312, 47.9%) of respondents post general photographs, such as those of a public figure, an image of nature, a city, or text with an image instead. Thus, they feel that they cannot post a photograph of themselves, but still seek to represent their personal identity. Table 4 shows that 180 (13.7%) participants post group photographs of family members that include themselves, which indicates that they would like to post personal photographs but are under pressure not to do so alone. As shown in Table 5, the main reason expressed by the 726 women who did not post their personal photograph on their social media accounts was fear that it would be misused (488/1312, 67.2%). This represents a major issue for women because they feel that it can affect their reputation. Table 5 shows that 112 (15.4%) respondents claim that they feel ashamed to show their photograph because Arab society is generally conservative, and women cannot express themselves even on social media. Seventy-four (10.1%) women revealed that their families do not allow them to post their personal photographs on social media. Their families fear the misuse of the photograph, which can affect reputation and honor.

**Table 4:** Types of images on social media accounts of Arab women (N=1312).

Type of image	Women, n (%)
Personal photograph	490 (37.3)
Photograph that contains me and my family	180 (13.7)
Photograph that contains me and my friends	14 (1.1)
General images such as those of a public figure, nature, a city, or a text with an image	628 (47.9)

**Table 5:** Reasons for not posting personal photographs on social media accounts.

Reasons	Women, n (%)
Fear of misusing my photograph by other people	488 (67.2)
My family does not allow me to post my photograph on my social media account	74 (10.1)
Society and cultural pressure	52 (7.1)
Feeling shameful	112 (15.4)
Total	726 (100)

#### Sexual Harassment via Social Media

This section examines the third research question, which addresses how Arab women face sexual harassment via social media. As shown in Table 6, at total of 494 (37.7%) participants stated that they encountered sexual harassment, and 508 (38.7%) stated that they somewhat encountered sexual harassment. Less than one-fourth (310/1312, 23.6%) of respondents claimed that they have not been sexually harassed via social media (Table 6). Thus, men are given yet another opportunity to engage in violence against women, beyond the public setting, workplace, and at home. Moreover, sexual harassment is often a disturbing experience. Survivors can be subjected to significant harm that not only inflicts emotional distress but also raises substantial problems in their family situations.

**Table 6:** Sexual harassment of Arab women via social media (N=1312).

Sexually harassed via social media	Women, n (%)
Yes	494 (37.7)
Somewhat	508 (38.7)
No	310 (23.6)

#### Women’s Legal Awareness of Sexual Harassment on Social Media

This section examines the fourth research question, which addresses legal awareness about sexual harassment on social media. Table 7 indicates that only half of the participants (646/1298, 49.8%) were aware that charges could be pressed against sexual harassment on the internet, and 222 (17.1%) were unaware. Approximately all 1264 (98.3%) participants would not take legal action against sexual harassers. As shown in Table 8, half (642/1298, 49.9%) of the participants did not want to take legal action against sexual harassers because they believe that it could affect their reputation. Another reason stated by many women (373/1298, 29%) was that they thought that the process of a sexual harassment lawsuit would take too long. Another 271 (21.1%) participants feared acts of revenge by sexual harassers. These findings indicate that women do not have complete confidence in judicial power and the police to protect them.

**Table 7:** Perceptions of sexual harassment as a punishable offense and willingness to press charges against harassers (N=1298).

Perception of sexual harassment being a punishable offence			Women, n (%)
**Do you think sexual harassers can be punished?**			
	Yes	646 (49.8)	
	No	222 (17.1)	
	Do not know	646 (49.8)	
**Would you take sexual harassers to court** **?**			
	Yes	22 (1.7)	
	No	1264 (98.3)	

**Table 8:** Reasons of not pressing charges against sexual harassers among Arab women (N=1286).

Reasons	Women, n (%)
It takes my time in the court	373 (29.0)
It affects my reputation	642 (49.9)
I fear that the accused will take revenge	271 (21.1)

### Results of Our Study Hypotheses

This section is focused on testing and interpreting the 8 study hypotheses. The first hypothesis predicted a positive correlation between the level of education of Arab women and posting their personal photograph on social media. Table 9 shows that this hypothesis (R_s_=–0.085; *P*<.001) is negatively correlated, which implies that women with lower education levels were more likely than those with higher education levels to post their personal photograph on social media.

The second hypothesis predicted that older Arab women were more likely to post the personal photograph on their social media accounts than younger women. This hypothesis (R_s_=–0.134; *P*<.001) was negatively correlated, similar to the first hypothesis. We assumed that educated and older women would understand and recognize problems such as sexual harassment better than relatively lesser educated and younger women and would therefore be more reluctant to post their photograph on social media.

The third hypothesis predicted that Arab women with higher levels of education would be less likely to face pressure for posting a photograph or a topic on social media than those who are relatively less educated because it is a common prediction that education empowers women to become more independent and respected among families, which this present study confirmed (R_s_=–0.076; *P*=.02).

The fourth hypothesis predicted that age is negatively associated with being condemned for posting a photograph or topic on social media. This hypothesis (R_s_=–0.053; *P*=.06) is not confirmed, which implies that Arab women are more likely being condemned for posting a photograph or topic on social media regardless of age.

The fifth hypothesis predicted a positive correlation between the education level of women and being sexually harassed via social media. This hypothesis (R_s_=0.083; *P*=.04) was confirmed, which implies that educated women face more sexual harassment via social media. It is assumed that they are more active in the public sphere, have wider relationships, and are more popular than lesser educated women, the latter probably spending more time within their families.

The sixth hypothesis predicted that age is negatively associated with being sexually harassed via social media, which implies that younger women more face sexual harassment. However, Table 9 shows that this hypothesis (R_s_=0.036; *P*=.003) was not confirmed, which implies that Arab women are likely to face sexual harassment on social media irrespective of their age. Table 9 shows that 494 (37.7%) participants stated that they encountered sexual harassment, and 508 (38.7 %) stated that they somewhat encountered it.

The seventh hypothesis predicted that the education level of women is associated with legal awareness about sexual harassment on social media, and this hypothesis (R_s_=0-125; *P*<.001) was confirmed.

The eighth hypothesis predicted that the education level of women is associated with a willingness to press charges against sexual harassers. This hypothesis predicted that educated women have more power and realize that the sexual harassment can be legally prosecuted and will be more likely to confront their harassers in court. However, this hypothesis (R_s_=0.014; *P*=.62) was not confirmed because the vast majority of participants (1264/1286, 98.3%) were not willing to press charges against their sexual harassers irrespective of their educational background (Table 7).

**Table 9:** The Spearman correlation model reports the associations between age and education levels of Arab women with posting their personal photographs on social media, being condemned for using social media, experiencing sexual harassment, and having legal awareness of sexual harassment on social media (N=1312).

Hypothesis	Variables	R_s_ value	Sig. (2-tailed) *P* value
1	Level of education is positively associated with posting a personal photograph on social media.	–0.085^a^	.002
2	Age is associated negatively with posting a personal photograph on social media.	–0.134^a^	<.001
3	Level of education is negatively associated with being condemned for posting a photograph or topic on social media.	–0.076^a^	.02
4	Age is negatively associated negatively with being condemned for posting a photograph or topic on social media.	–0.053	.06
5	Education level of women is positively associated with being sexually harassed via social media.	0.083^a^	.06
6	Age of women is not associated with being sexually harassed via social media.	0.036	.19
7	Education level of women is associated with legal awareness of sexual harassment on social media.	0.125^a^	<.001
8	Education level of women is associated with willingness to pressing charges against sexual harassers.	0.014	.62

^a^The correlation is significant when R_s_ values range from –1 to +1.

## Discussion

### Principal Findings

There is a lack of studies examining the positive and negative aspects of social media in the lives of Arab women [39]. Previous studies at the global level have referred to a number of positive aspects of social media platforms, such as their role in facilitating interaction and participation and disseminating information [22-24]. Other studies have highlighted the positive aspects of social media in boosting the feminist movement [20], understanding women’s issues [18], and raising awareness about sexism and misogyny [21]. In this study, only 122 (9.3%) participants indicated that they used social media for promotions and personal branding (Table 3) [44,45]. These positive aspects of social media help women to become entrepreneurs and launch web-based businesses and other positive ventures. Besides this, 120 (9.1%) participants indicated that social media is an entertainment platform for them and social media lets them access global culture, even if they live in a relatively conservative society (Table 3). Fear of image-based abuse, blackmailing, and interference in their private lives were reasons that motivated half of the participants to refrain from posting personal photographs on their social media profiles. For instance, Table 4 indicates that most Arab women were reluctant to post their personal photographs on their social media accounts and only approximately one-third (490/1312, 37.3%) did so. Alternatively, they posted images of local or international celebrities, nature, or family group photographs, which are more difficult to alter or use maliciously. This situation counters the liberal notion that women can express themselves on social media and use social media as a platform to play influential roles in society [1-3,16]. Even though laws in many Arab countries clearly define harassment on social media as a crime, many women encounter some level of sexual harassment (Table 6) irrespective of their age. Although some women knew that this is a crime (Table 7), they indicated that they would not come forward and press charges against their harassers for several reasons, including bringing dishonor upon their families, the time-consuming nature of litigation, and the fear of revenge.

### Limitations

The majority of previous studies have focused on individual Arab countries rather than adopting a regional approach, and have used descriptive rather than data-based approaches. This does not allow for extensive comparison with this study’s outcomes. Regarding data collection, doing so in-person across all Arab countries representationally is difficult and costly. This was also prevented by the COVID-19 pandemic in 2020. Therefore, this study adopted a survey method using a web-based questionnaire administered through Google Forms (N=1312) with responses from Arab women aged ≥15 years from all Arab countries. More extensive data collection would further improve our results.

### Conclusions

This study predicts that women’s endurance of the vast majority of sexual harassment without taking legal action against sexual harassers could motivate harassers to continue engaging in abusive behavior. This harms women and creates a climate of intimidation and repression in social and economic life, particularly among young women, as this study shows that age is negatively associated with being condemned for posting a photograph or topic on social media (Table 9). Conversely, the education level of women plays a positive role in independence within the family. Our third hypothesis predicts that Arab women with lower levels of education might face more pressure for posting a photograph or topic on social media than those with higher education levels. This study found that young women and the relatively lesser educated are more likely to face abuse. This leads to several issues including lower self-esteem and hesitancy in looking for a job, feelings mistrust and fear, cynicism, anxiety, depression, and sleep disorders. These issues constrain women from using social media and some of them think about disengaging from social media. This issue needs governments’ consideration to develop laws to reduce violence and empower women to take legal action. Hence, future studies should address the violence against women with regard to legal and psychological perspectives.
